# High-dose of intravenous immunoglobulin modulates immune tolerance in premature infants

**DOI:** 10.1186/s12887-018-1055-5

**Published:** 2018-02-21

**Authors:** Pin Liu, Lijun Li, Panpan Fan, Junwen Zheng, Dongchi Zhao

**Affiliations:** Pediatrics and Neonatology Department, Zhongnan Hospital of Wuhan University, Donghu road 169, Wuhan, 430071 China

**Keywords:** Intravenous immunoglobulin, FoxP3+ Treg, Preterm, Cytokines

## Abstract

**Background:**

Intravenous immunoglobulin (IVIG) is commonly used to improve the immunomodulatory effects, although its regulatory effect on premature Treg cells is unclear. The purpose of this study is to study the effect of high dose of IVIG (HD-IVIG) on Treg cells expression and cytokine profile in premature birth.

**Methods:**

Fifty-two premature infants were enrolled in this study and thirty-one premature infants who were suspected to have intrauterine infection received HD-IVIG (1–2 g/kg) at the first day of birth; the remaining 21 premature infants were assigned as the control group. The peripheral blood CD4 + T and foxp3+ Treg cells were checked by flow cytometry, and cytokine concentrations were detected by cytometric bead array.

**Results:**

With the gestational age growth, peripheral blood CD4 + T and foxp3+ Treg cells of prematurity gradually declined from 50% to 35% and from 8% to 6%, respectively. Meanwhile, HD-IVIG increased the percentage of CD4 + T and foxp3+ Treg cells compared with their baseline levels (*p* < 0.001). HD-IVIG demonstrated different regulating effects on cytokines secretion, increased IL-17 and TGF-β, and inhibited IL-6 secretion.

**Conclusion:**

Our results demonstrated that HD-IVIG not only enhanced the premature immune tolerance, but also suppressed the excessive inflammation response mediated by IL-6.

**Trial registration:**

This study was under the clinical study registration (ChiCTR-ORC-16008872, date of registration, 2016–07-21).

## Background

Early-onset infection is a serious complication of premature infants, and it is still a main cause of morbidity and mortality in neonates [1]. Premature infants are more prone to develop sepsis because of their immature immune response [2]. Approximately 11–20% of all births worldwide were diagnosed as preterm, and 37% of those premature infants were caused by infections [3].

Premature infants are at higher risk than term infants to develop infections, with an incidence of 16.6%, and 90% of the infections occurred during the first 72 h of life [4, 5]. Because of systemic infection in preterm infants, even with appropriate antibiotic treatment, adjunctive therapies have been recommended to improve the outcomes of preterm infants [6]. Intravenous immunoglobulin (IVIG) is a polyclonal immunoglobulin G preparation with widely immunomodulatory properties. IVIG can increase the number and the suppressive capacity of regulatory T cells (Treg), a sub-population of T lymphocytes with CD4 + CD25 + Foxp3+ phenotype, which is essential for immune homeostasis. In addition, IVIG also alters the function of immune cells, cytokine and chemokine networks, and the orientation of T lymphocytes [7], which prevent patients from suffering from secondary harm caused by the over-response inflammation. Usually, IVIG is used at a ‘replacement dose’ (400–600 mg/kg) in antibody deficiencies and at a high dose (1–2 g/kg) as an ‘immunomodulatory’ agent in immune and inflammatory disorders.

The Foxp3+ Treg cell is a sub-population of CD4 + T, which plays a critical role in peripheral tolerance and the control of immune responses to pathogens [8], and it participates in abrogating immune responses, thereby preventing exacerbated and potentially deleterious immune activation [9]. Premature neonates persistently have a higher proportion of CD4+ Treg cells and similar interferon gamma (IFN-γ) compared with term neonates [10]. The Treg pool of premature infants could be altered by prenatal exposure to inflammation and chorioamnionitis, which lead to functional decrease in Treg cells [11]. However, whether IVIG infusion affects Foxp3+ Treg cells differentiation in premature infants is unclear, and the clinical implications need further clarification.

In the present study, we measured the effect of the high-dose IVIG (HD-IVIG) on late and moderate preterm Treg cell differentiation and pro-inflammatory cytokines secretion.

## Methods

### Subjects description

This study was conducted from July 2015 to December 2016 in Zhongnan Hospital of Wuhan University, China, under the clinical study registration (ChiCTR-ORC-16008872). Fifty-two infants were consecutively admitted to our neonatal care intensive unit (NICU) with a gestational age (GA) between 32 and 36 weeks (GA 32 ^0/7^–36 ^6/7^ weeks). The reason for preterm is shown in Table 1. All the premature patients suspected with sepsis were admitted to the NICU and recruited into the study, with the following exceptions: premature babies with congenital malformations, autoimmune diseases, those whose date of birth was missing or uncertain, and when the guardian of the premature infants was unwilling to participate in the study.

**Table 1:** Characteristics of the IVIG-treated group and the control group

	IVIG	Control	*P* value
Patients	31	21	
Gender			
Male	16	12	0.7626
Female	15	9	
GA (w, median)	33.5 (32.0–36.1)	34.1 (30.1–36.3)	0.1082
BW (g, median)	2025 (1520–2670)	2096(1540–2810)	0.4923
Mode of delivery			0.755
Vaginal	7	5	
Cesarean	24	16	
Preterm reasons			
PROM	19	15	0.194
PIH	9	4	0.064
PP	3	2	1
NCPAP			0.127
Yes	16	9	
No	15	12	
Apgar at 1 min	7.43	7.85	0.1263
Apgar at 5 min	8.71	9	0.2421
Hospital stay (d)	15.2	12.67	0.0549
Prognosis	Cure	Cure	

*GA* Gestational age, *BW* Birth of weight, *PROM* Premature rupture of membranes, *PIH* pregnancy-induced hypertension, *PP* placenta previa, *NCPAP* nasal continuous positive airway pressure

### High-dose of IVIG treatment protocol

Fifty-two suspected sepsis premature infants were enrolled in this study and divided into the IVIG infusion group and the control group. All patients received the prevention antibiotics therapy, among them, thirty-one premature infants received HD-IVIG (1-2 g/kg) at the first day of birth, and the remaining of 21 premature infants were given an equal volume of 5% glucose.

### Ethics statement

This study was approved by the Ethics Committee of Zhongnan Hospital, Wuhan University (protocol 2,015,019), where the study was performed and all guardians signed the inform consent for publication. All data and materials are availability.

### Blood samples

Peripheral blood was collected twice: in the first hour after birth before IVIG infusion, and on the fifth day after the infants were admitted to the NICU. IVIG infusion was performed on the first day of hospitalization. A total of 2 ml of blood was collected and mixed in EDTA tubes. Plasma extracted from the blood was stored in − 80 °C for the subsequent detection of cytokines. Peripheral blood mononuclear cells (PBMCs) were isolated from the whole blood by density gradient sedimentation according to the manufacturer’s instructions (Lymphocyte separation medium, MP Biomedicals, Burlingame, CA, USA).

### Flow cytometry

For the analysis of Treg cells, PBMCs were stained with anti-CD4-FITC and CD25-APC cocktail monoclonal antibodies (eBiosciences, San Diego, CA, USA) at room temperature in the dark for 20 min. After washing, the cells were resuspended in fixation/permeabilization (eBiosciences) working solution and incubated at 4 °C in the dark for 30 min. Intracellular staining was then performed with anti-Foxp3-PE and isotype control (eBiosciences) in permeabilization buffer, according to the manufacturer’s instructions. After staining, the cells were washed and resuspended in phosphate-buffered saline for measurement by BD FACSVerse flow cytometry (BD Biosciences, San Jose, CA, USA). Data were analyzed by FlowJo data analysis software (FlowJo, LLC, Ashland, OR, USA).

### Cytometric bead array (CBA)

The plasma for the detection of TGF-β1 required pre-acidification and neutralization (BD™ Human TGF-β1 Flex Set, BD Biosciences, San Jose, CA, USA), whereas the plasma for detection of interleukins (IL) such as IL-2, IL-4, IL-6, IL-10, TNF-β, IFN-γ, and IL-17A (BD™ CBA Human Th1/Th2/Th17 Cytokine Kit, BD Biosciences, San Jose, CA, USA) do not need any pre-treatment. All the cytokine levels were measured by CBA technique on FACSVerse cytometry according to the manufacturer’s instructions. Data were analyzed by FCAP Array software (BD Biosciences, San Jose, CA, USA).

### Statistical analysis

Analyses of variance were measured by the Wilcoxon signed rank test. A non-parametric student t-test (Mann-Whitney) was used to compare the differences between subgroups. Data were obtained using GraphPad Prism version 5.0. Results are presented as mean ± SD. *P* < 0.05 was considered as significant difference.

## Results

### General information of subjects

Of 52 cases of preterm neonates, 31 received IVIG and 21 were assigned as controls. The clinical features are shown in Table 1. There was no difference in gender, gestation age, body weight in birth, the mode of delivery, and preterm birth reasons (*p* > 0.05). All preterm infants were 32–36^+ 6^ weeks in gestation age. There were no deaths among the infants during the period of the hospital stay and the first half-year of their life. Most of the preterm infants were premature rupture of membranes (PROM) in the IVIG (60.7%) group and the control group (83.3%).

### Gestational ages correlated to preterm CD4 + T subgroup differentiation

**Fig. 1:**
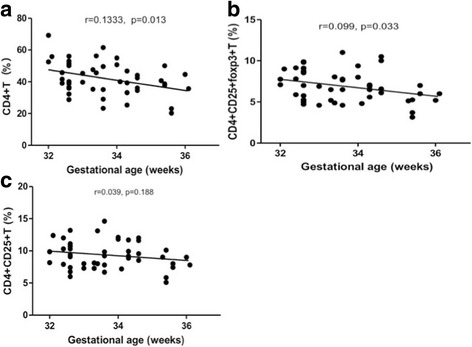
Percentage of CD4 + T, Foxp3+ Treg, and CD25 + T cells in preterm infants and the correlates with gestational age. **a**, **b**, and **c**, scatter plot graph showing a negative correlation between GA (X-axis, in weeks) and cell distribution percentage (Y-axis). Pearson’s correlation test analyses

The CD4 + T cells of all premature births were detected by a flow cytometric measurement. Pearson analyses demonstrated that the CD4 + T cells distribution was negatively correlated to gestational ages (Fig. 1a), and the same trend was observed in CD4 + CD25 + Foxp3+ Treg cells (Fig. 1b). There was a significant correlation between GA and newborn CD4 + T cells subsets abundance. These results imply that, along with the fetus development, the CD4 + T and CD4 + CD25 + Foxp3+ Treg cells were reduced. CD4 + CD25 + T cells were also reduced during this period, but there was no significant relationship between these cells proliferation along with GA (Fig. 1c). These results demonstrated that Foxp3+ Treg cells were reduced in more proportions in CD4 + CD25 + T subgroup along with GA.

### HD-IVIG upregulated the proportion of CD4 + T subsets in premature infants

**Fig. 2:**
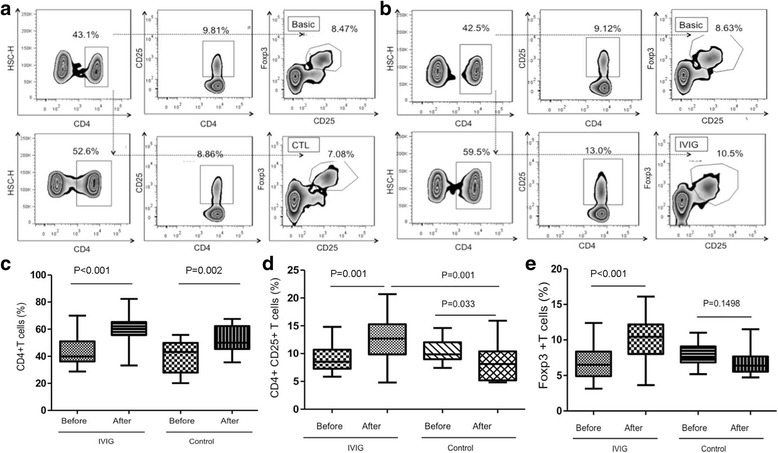
Representative gating scheme to identify regulatory and non-regulatory CD4+ T cells by flow cytometry. First, using the side scatter (SSC) and forward scatter (FCS) gate lymphocyte subgroup. Next, CD4+ T cells were based on the CD4/SSC strategy. Further sub-classified as CD4 + CD25 + Foxp3+, Tregs were identified by respective isotype control. **a** percentage of cell populations of the control group. **b** percentage of cell populations of the IVIG infusion group. Percentage of CD4 + T, CD4 + CD25+, and Foxp3+ Treg cells in the IVIG infusion group and the control (CTL) group. The percentage of CD4+ T (2**c**), CD4 + CD25+ (2**d**), and Foxp3+ Treg (2**e**) cells was measured with flow cytometry at times before and after IVIG infusion. Patients were grouped into IVIG infusion and control (CTL) groups. Means ± SD are shown

To evaluate the high-dose of IVIG effect on premature immune cells proliferation and differentiation, the CD4+, CD4 + CD25+, and CD4 + CD25 + Foxp3+ cells in the first day of birth were detected by cytometry methods, and the data are shown in Fig. 2a and b, respectively. The CD4 + T cells increased in both the IVIG infusion and control groups (Fig. 2c), whereas the CD4 + CD25 + T and Foxp3+ Treg cells increased in the IVIG infusion group and decreased in the control group significantly (Fig. 2d, e). The percentage of CD4 + T and CD4 + CD25 + T cells increased 50%, and the Foxp3+ Treg cells increased 60% compared with their basic levels in IVIG-treated premature babies, whereas both CD4 + CD25 + T and Foxp3+ Treg cells decreased in the control group from. These results implied that despite the percentage of CD4 + T cells increasing physiologically in the first 5 days after birth, the CD4 + CD25 + T and Foxp3+ Treg cells did not increase consistently with CD4 + T cells. In contrast, the CD4 + CD25 + T and Foxp3+ Treg cells obtained a significant increase after IVIG infusion.

### HD-IVIG modulated premature cytokines expression profile

**Fig. 3:**
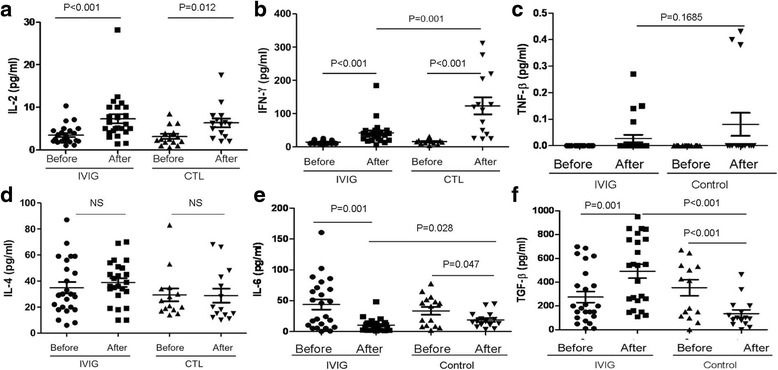
Concentration of novel cytokines (3**a**, IL-2, 3**b**, IFN-γ, 3**c**, TNF-β, 3**d**, IL-4, 3**e**, IL-6 and 3**f**, TGF-β) in the peripheral blood of IVIG infusion and control neonates was determined by CBA. Data are represented as mean ± SD. Statistical analysis was performed using Student’s t-test at 95% confidence level; *p* values < 0.05 are considered statistically significant

The serum cytokines were measured under a different environment. The IL-2 expression increased in 5 days in both the IVIG infusion and control groups compared with their basic levels (Fig. 3a), and there was an increase in IFN-γ in the IVIG and control groups (Fig. 3b). Compared with the IVIG infusion group, the control group expressed five-fold of IFN-γ more than the IVIG infusion group. Both the IVIG and control groups showed a slight increase in TNF-β (Fig. 3c), but there was no significant change compared with their basic levels.

**Fig. 4:**
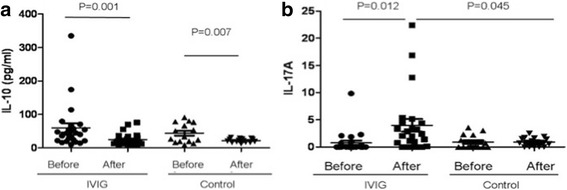
Concentration of novel cytokines (4**a**, IL-10 and 4**b**, IL-17A) in the peripheral blood of IVIG infusion and control neonates was determined by CBA. Data are represented as mean ± SD. Statistical analysis was performed using Student’s t-test at 95% confidence level; *p* values < 0.05 are considered statistically significant

There was no difference in IL-4 induction between the IVIG infusion and control groups compared with their basic levels, whereas IL-6 concentration decreased in both the IVIG and control groups (Fig. 3e). In contrast, IVIG results in IL-6 being significantly reduced than the control group. TGF-β is the Foxp3+ Treg cells stimulator, whereas IL-6 combined TGF-β induces Th17 cell differentiation, and IL-6 suppressed Treg orientation induction. Compared to the control group, in which TGF-β was reduced in 5 days compared with their baseline level, IVIG infusion significantly increased TGF-β secretion. On the basis of the result of IVIG regulating TGF-β and IL-6 secretion, the increase in Foxp3 Treg cells could affect Th17 cell function. Next, the serum IL-10 and IL-17A concentration were detected. The IL-10 concentration decreased in both the IVIG and control groups (Fig.4a); unexpectedly, however, the IL-17A increased after IVIG infusion (Fig.4b). These results suggested that premature naïve immune cells phonotype might not be consistent with their functional development.

## Discussion

In the present study, we report the effect of HD-IVIG on the phenotypic and functional consequences of CD4 + T subsets in premature infants. The CD4 + T and Foxp3+ Treg cells decreased along with the pregnancy term in preterm birth, whereas the HD-IVIG promoted the CD4 + T and Foxp3+ Treg cell differentiation in several days after birth. IVIG infusion played a sophisticated role in regulating CD4 + T cell subsets distribution and cytokine distribution, which improved premature immune tolerance and suppressed undue inflammation response.

To date, IVIG has been widely used in pediatrics for immune disorder diseases. Different from the moderate dose of substitution, high-dose IVIG increases the concentration two-fold more than the physiological immunoglobulin level [12], which functions as immune modulation [13]. In preterm infants, the pathogens usually cause severe systemic symptoms through the bias of immune function, especially the over power of type II cytokines [14]. Immune bias causes dysregulation of other systems, leading to multi-organ dysfunction and failure. Thus, the aim of treatment in severe infection is to control the hemodynamic impairment and organ dysfunction [15]. In addition, providing immunotherapy to restore immune homeostasis is proving to be an important causal approach to modulate and affect the inflammatory process. However, a multicenter research report showed that therapy with IVIG had no effect on the outcomes of suspected or proven neonatal sepsis [16]. Despite those negative results in neonatal sepsis trials, the high dose of IVIG is still commonly used as immune regulation during severe preterm infection [17–19], and additional mechanisms need to be analyzed [20, 21].

Many mechanisms explaining the immunoregulatory actions of IVIG have been postulated, including the blockade of activating FcγR on antigen-presenting cells, interference with cytokine production, inhibition of cell activation, or induction of apoptosis in a variety of immune cells [22]. Among those, a key factor in immune modulation is the ability to counter inflammatory responses with regulatory cells. It is very important that HD-IVIG increases the percentage of Foxp3 + T cells in premature infants, because the Foxp3 + Treg cell plays a role of immune tolerance, and the infusion of IVIG reduced the inflammation to external stimulation in severe sepsis. HD-IVIG infusion actually plays a critical role both in peripheral tolerance and control of immune responses to pathogens. Premature infants could benefit from avoiding an undue inflammation response caused by severe infection [23].

Our results shown that increased TGF-β and decreased IL-6 are consistent to the abundance of Foxp3 Treg cells under IVIG infusion. HD-IVIG not only induced the premature Foxp3+ Treg cells expression, but also improved Th17 cell-related functions. Severe sepsis is a condition of cytokine-mediated unbalanced immune homeostasis. In this study, we found that HD-IVIG modulated cytokines distribution in a complex manner in premature infants, which regulated cytokines function by suppressing IFN-γ and IL-6 induction, but promoted IL-2 and TGF-β secretion. Because TGF-β determines CD4 + CD25 + T cell orientation to the Foxp3+ Treg, whereas IL-6 induces Th17 differentiation, these cytokine expression profiles were consistent with the increased Foxp3+ Treg cells under IVIG infusion. It has been demonstrated that an in vitro culture of IVIG with T cells led to increase in intracellular TGF-β, IL-10, Foxp3 expression and improvement in their suppressive functions when co-cultured with effector T cells [10].

IL-10 concentration decreased in both the IVIG and control groups, which were consistent with the IFN-γ increase, and IL-17 actually increased through IVIG intervention. This could be attributed to two reasons: the premature infant’s immature naïve T cells whose function did not develop with their surface markers, and the small samples. The temporary immune tolerance could be a benefit in premature infants for restoring system homeostasis; however, the cytokine profile changes, such as increasing TGF-β and decreasing IL-6, which also could be correlated with the subsequent bronchopulmonary dysplasia [24], which need additional study and documentation. The limitations of this study includes the fact that we did not observe a time period effect of IVIG on Treg cell distribution and cytokine expression profile, which needs further study in the future.

## Conclusions

HD-IVIG increased the premature percentage of Foxp3 + T cells, modulated cytokine expression by promoting TGF-β and IL-17, and inhibited IL-6. Preterm infants could benefit from avoiding increased inflammation and restoring unbalanced immune homeostasis situation.

## Abbreviations

GA: Gestational age
HD-IVIG: High dose of IVIG
IFN-γ: Interferon gamma
IL: Interleukins
IVIG: Intravenous immunoglobulin
NICU: Neonatal care intensive unit
PBMCs: Peripheral blood mononuclear cells
PROM: Premature rupture of membranes
Treg: Regulatory T cells
